# The Spontaneous Imbibition of Micro/Nano Structures in Tight Matrix and the Influence on Imbibition Potential

**DOI:** 10.3390/mi11090794

**Published:** 2020-08-21

**Authors:** Caoxiong Li, Chenggang Xian, Jun Wang, Dandan Geng, Yinghao Shen

**Affiliations:** 1 Unconventional Oil and Natural Gas Institute, China University of Petroleum, Beijing 102249, China; licaoxiong@cup.edu.cn (C.L.); shenyinghao@126.com (Y.S.); 2 State Key Laboratory of Petroleum Resources and Prospecting, Beijing 102249, China; 3 The No. 7 Oil Production Plant Company, Changqing Oilfield, CNPC, Xi’an 710000, China; spirit-jun@hotmail.com; 4 The No. 6 Oil Production Company, Daqing Oilfield, CNPC, Daqing 163114, China; gengdandan@petrochina.com.cn

**Keywords:** tight matrix, water imbibition, pore structure, fractal theory

## Abstract

Tight matrix has relatively low permeability and porosity, with abundant micro/nano pores. The capillary force in these pores are relatively strong, making the wetting liquid easier to be imbibed in the matrix. This process is called spontaneous imbibition. The complexity of pore structure is identified as one of the key factors influencing the imbibition process, but the detailed mechanism is not clear. Thus, in this work, a method is proposed to evaluate the influence of pore structure on imbibition process. Pore structure has fractal properties in a specific scale. By using the fractal theory, an imbibition model is provided to analyze the influence of microscopic structures on spontaneous imbibition, considering the pore size distribution and pore connectivity. Also, based on this model, the influencing factors on dimensionless imbibition and diffusion rate are discussed. Results show that the pore structure has more branches, larger and shorter sub-throats has higher chance to gain a high imbibition rate. Finally, a 3D imbibition parameter cube is constructed to determine the parameter combinations in favor of strong water diffusion potential. By utilizing the analysis method based on the fractal theory, we can effectively evaluate the imbibition potential. It is helpful to provide a guidance to evaluate the water imbibition to gas production.

## 1. Introduction

Large volume hydraulic fracturing has often been used to enhance production by creating artificial fracture systems in tight reservoir [1]. In this process, the spontaneous imbibition of fracturing liquids into the matrix exerts a microscopic displacing effect, thereby enhancing pore pressure. Permeability is relatively low for tight reservoir, whereas imbibition effect is strong. When the imbibition rate or imbibition potential is strong enough, gas recovery can be improved by self-unblock effect or imbibition effect [2]. The imbibition rate or imbibition potential critically influences the imbibition intensity, which is related to the complexity of the pore structure.

Imbibition can be classified as either co-current or counter-current imbibition. Co-current imbibition can be simulated in the capillary tube model derived by Scheidegger [3]. Since Lucas [4] and Washburn [5] gave the initial expression of the relation between the imbibition distance of liquid and time in 1921, numerous studies have extended their work. Martic et al. declared that the contact angle in the Lucas–Washburn equation should be dynamic [6]. Cupelli et al. posited that the inertia of the liquid should be considered [7]. Fries and Dreyer investigated for an analytic solution for capillary rise in inclined tubes [8]. Cai et al. provided the analytical expressions for the height and mass of imbibed liquid during capillary rise process [9]. Yu et al. introduced fractal method to describe the tortuousness of capillary [10,11]. Li et al. built a capillary displacement model for tight matrix considering the dynamic effect [12]. Wang et al. provided self-similar analytical solution of spontaneous imbibition in porous media, considering the influence of capillary pressure [13]. Zhou et al. evaluated the complex gas/water-oil displacement system in tight reservoir based on experiments [14]. However, capillary tube models commonly ignored the complexity and connectivity of the pore structure. For this reason, these models should be extended to simulate the imbibition process. Recently, many researchers have provided a series of new models for spontaneous imbibition, Li et al. summarized some commonly used models and methods to evaluate the imbibition process in tight matrix [15].

Considering the variety of pores, imbibition curves have different shapes. Imbibition curves are affected by complex structure of pores. It is a hard work to reconstruct real pore spaces for tight matrix. Luckily, pore structures have fractal properties in a specific scale [16,17,18]. A fractal tree-type network system model provides an effective way to simplify pore space of tight matrix. In fractal network system, the diameter and length of the tubes in each branch are determined according to fractal law, and their ratio between neighboring levels is deterministic, thus demonstrating the system’s self-similar (i.e., fractal) properties. Many studies have explored such a network system. Pence et al. computed the pressure and temperature distributions of this fractal-like branching network [19,20], and Xu studied the heat conduction and permeability of this network system [21,22].

Pore structure greatly affects imbibition rate and imbibition potential, but the mechanism is still unclear. In this work, an imbibition model that combines liquid imbibition property and fractal pore structure is derived to simulate the imbibition process in porous media. This model focuses on the relationship between pore structure and imbibition process. An analytical expression is derived for the imbibition potential. A chart based on this expression is established. This chart directly shows the pore structures guaranteed to have imbibition potential.

## 2. Fundamental Features of the Fractal Pore Structure and Network Model

**Figure 1:**
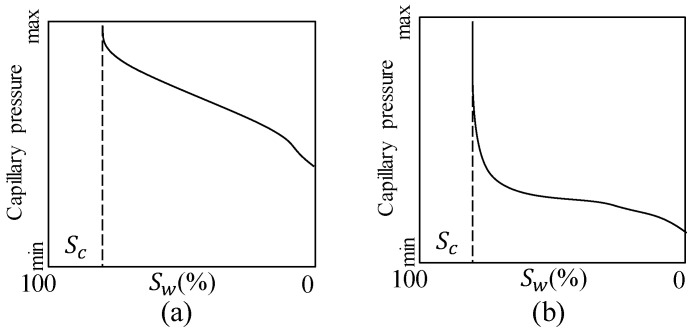

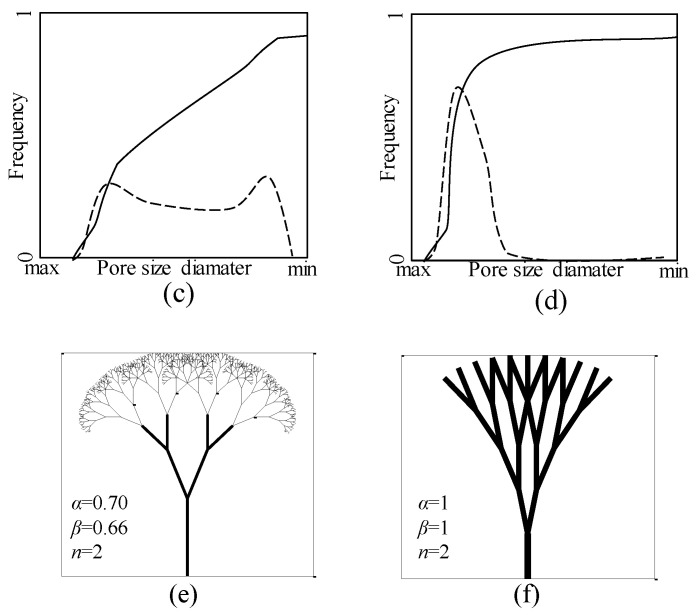
Relationship between pore structure and fractal network in tight and normal matrix. (**a**,**c**) show the schematic of mercury injection features and pore size distribution for typical tight matrix, (**b**,**d**) show the schematic of mercury injection features and pore size distribution for normal matrix. (**e**) represents the fractal network for tight matrix. (**f**) represents the fractal network for normal matrix which has less branches and larger pores.

Tight matrix is special, particularly in pore structure. The pores in tight matrix are typically much smaller than normal formation; thus, tight matrix has a higher threshold pressure in the mercury injection curve. Moreover, it has more branch throats and micro-nano sub-pores. The schematic of mercury injection features and pore size distribution are expressed in Figure 1a,c (tight matrix), Figure 1b,d (normal matrix). As discussed, pore structures has fractal properties in a specific scale and can thus be described as a special fractal network. This model has the same pore size distribution as the target pore structure. It can also represent the differences on pore structure between tight and normal matrix, as shown in Figure 1e,f. The figures show that tight matrix has much more branches and smaller pores than normal one.

On the basis of the characteristics of the fractal pore structure model, the tree structures are generated by repeating themselves that follow a properly designed algorithm, resulting in an increasing number of channels with smaller diameters (Figure 1). In this work, the fractal model is used to simulate the imbibition process. Water is absorbed into the main channel and then asymptotically flowed into the branches. For simplification, we assume that the branches in model are a smooth cylinder, and the effects of both the wall layers and the clay minerals are ignored. Flowing status is simplified as laminar flow of newton fluid. In this model, k represents the branching level, which refers to the diameter range of the pore that displays fractal properties (e.g., the main channel is level 0, *k* = 0). k also represents the imbibition front movement as water absorbed in thinner throats. A typical branch at level k has length $l_{k}$ and diameter $d_{k}$. The ratio of the adjacent level in length and diameter are α and β, we introduce these factors as [23]

(1) $$\alpha =l_{k+1}/l_{k},\;\beta =d_{k+1}/d_{k}$$

(2) $$l_{k}=l_{0}\alpha^{k},\;d_{k}=d_{0}\beta^{k}$$

where α and β represent the distribution of tubes in length and diameter, respectively. Every channel is divided into n branches, which represent the connectivity of the throat in the rock. A certain level k that has N branches can be represented as:

(3) $$N=n^{k}$$

According to the fractal characteristics of the structure [23], we have:

(4) $$n=\alpha^{-D_{l}}=\beta^{-D_{d}}\;\mathrm{or}\;D_{l}=-\mathrm{lg}n/\mathrm{lg}\alpha ,\;D_{d}=-\mathrm{lg}n/\mathrm{lg}\beta$$

where $D_{l}$ and $D_{d}$ represent the fractal dimensions of the throat length and the diameter. According to the conclusions of Xu [21], fractal dimension varies from 1 to 3.

According to Shen et al. [24], in general, the imbibition process can be divided into at least two stages. The first stage is called the imbibition stage, at which water is imbibed into the main pore–throat structures. Based on Hu [25] and K.Makhanov [26,27], the imbibition curve at this stage can be represented as $M(t)=A_{i}t^{n_{i}}$. $A_{i}$ denotes the imbibition rate, which reflects the imbibing speed of the rock at the initial stage; this variable is related to pore diameter, contact angle, viscosity, surface tension, and so on. $n_{i}$ represents the imbibition index, which mainly reflects pore connectivity and complexity; this variable is related to pore tortuosity and pore size distribution. M denotes imbibition mass, which mainly reflects water absorbing capacity.

The second stage is called the diffusion stage, which represents the potential of water absorbed in micro-nano sub-structures to unlock blocked gas and unlock the blocked main pore–throat structure. The diffuse ability of liquid in matrix is important. Diffusion ability is defined as the ratio of imbibed liquid mass in diffusion part to ultimate imbibed liquid mass, which can be derived from imbibition curve. We have defined a parameter *F_d_* to represent the liquid diffuse ability from imbibition curve, which is given by [24]:

(5) $$F_{d}=\frac{M_{d}}{M_{max}}$$

where *M_d_* is the imbibed liquid mass in diffusion part, *M_max_* is the ultimate imbibed liquid mass. The diffusion ability cannot be disregarded in tight rocks, particularly in tight matrix, because of this rock’s huge number of sub-structures, which exerts microscopic gas displacing effects that enhance gas production.

## 3. Co-Current Imbibition Based on the Fractal Model

**Figure 2:**
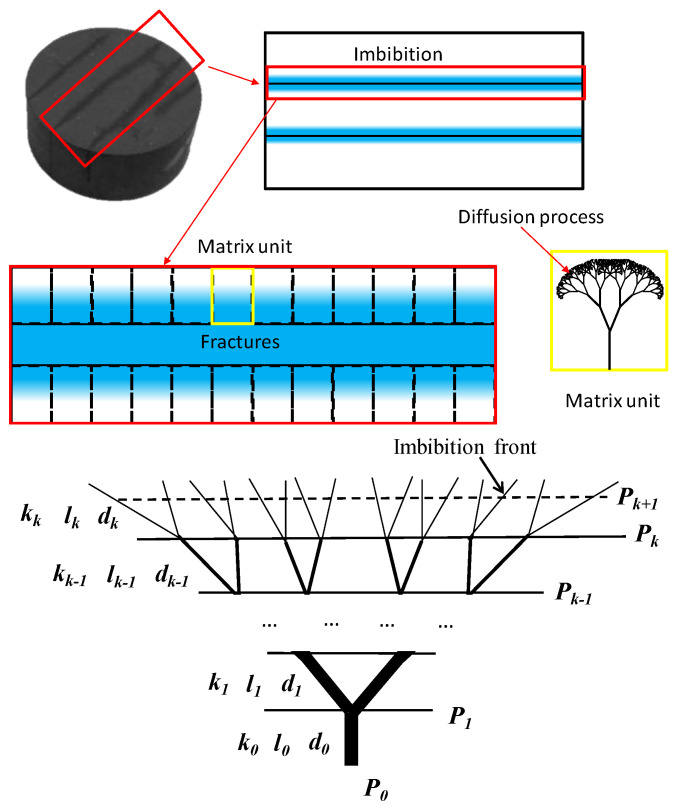
Schematic of the equivalent model of the fractal network system.

The model discussed in the previous section provides a way to simulate co-current imbibition in pore space, which is simplified as a fractal network system model by pore size distribution (Figure 2). In the network, each tube is regarded as a cylinder, branching sub tubes in each branching level. Length and diameter of each tube follow fractal law, as discussed in last section. Water is absorbed from left to right, driven by capillary pressure. Equivalent flow resistance method is introduced. Flowing resistance in every branching level is combined as the flowing resistance. Capillary pressure acts as the driving force.

With the imbibition of liquid, flowing resistance increases. In each branch level, capillary pressure also changes with the number and diameter of sub tubes. But one thing is for certain, the total imbibition rate $Q_{k}$ in network is controlled by capillary pressure and flowing resistance. In this model, we used a dimensionless parameter ${Q_{k}}^{+}$ to evaluate the imbibition potential as [28]:

(6) $${Q_{k}}^{+}=\bar{Q_{k}}/\bar{Q_{0}}$$

where $\bar{Q_{k}}$ is the imbibition rate in diffusion process (kth network), $\bar{Q_{0}}$ is initial imbibition rate, representing the imbibition rate in initial process (1th network). The imbibition rate is defined as the average volume flow rate in the model, such as $\bar{Q}=d(M/\rho )/dt$. The dimensionless parameter ${Q_{k}}^{+}$ in each branch level is set by the ratio of average flow rate in kth level to average flow rate in initial level, to make things clear and focus on the influence of structural factors on imbibition process. $Q_{k}{}^{+}$ can be expressed as Equation (8), detailed deduction process is shown in Li et al. [28].

(7) $$Q_{k}{}^{+}=\frac{\bar{Q_{k}}}{\bar{Q_{0}}}=\frac{1}{\beta^{k}}\frac{1-\alpha /(n\beta^{4})}{2-{\left[\alpha /(n\beta^{4})\right]}^{k}[1+\alpha /(n\beta^{4})]}$$

Or it can be expressed with the fractal dimension of throat length and diameter distribution as [28]:

(8) $$Q_{k}{}^{+}=\frac{\bar{Q_{k}}}{\bar{Q_{0}}}=n^{-k/D_{d}}\frac{1-n^{4/D_{d}-1-1/D_{l}}}{2-n^{4k/D_{d}-k-k/D_{l}}(1+n^{4/D_{d}-1-1/D_{l}})}$$

The liquid in this model is water, and the flowing behavior follows the Hagen-Poiseuille equation. Although the slip exists in both gas phase [29] and liquid phase [30], we ignored its effect and focus on the influence of pore structure. When the imbibition front is at k th level, the imbibition mass $M_{k}$ can be determined as:

(9) $$M_{k}(t)=\rho (\sum_{i=0}^{k-1}V_{i}+V_{k}(t))=\rho \left[\frac{l_{0}\pi d_{0}^{2}}{4}\frac{1-{\left(\alpha \beta^{2}n\right)}^{k}}{1-\alpha \beta^{2}n}+\sqrt{\frac{2A}{C}t+\frac{B^{2}}{C^{2}}}-\frac{B}{C}\right]$$

where $A=P_{k+1}-P_{0}$, $B=\frac{128\mu l_{0}}{\pi d_{0}^{4}}\frac{1-{\left[\alpha /(n\beta^{4})\right]}^{k}}{1-\alpha /(n\beta^{4})}$, $C=\frac{128\mu }{\pi n^{k}d_{0}^{4}\beta^{4k}}\frac{1}{\pi d_{k}^{2}n^{k}/4}$. $P_{k+1}$ and $P_{0}$ are the capillary pressures, μ is liquid viscosity. As shown in Equations (5) and (6), $Q_{k}{}^{+}$ is only related to the structure of the model, making the influence of pore structure on imbibition process more easy. To sum up, we can generate a fractal network model to simulate and simplify pore structures of real samples, with the same pore size distribution between fractal model and real samples. Then fractal parameters are obtained and are helpful to evaluate imbibition potential of real rocks.

## 4. Experiments

The samples are from the formation in Yanchang Shale Gas Field, Chang-7 Continental Shale Formation of Triassic in Erdos Basin. The Chang-7 shale sample is the fresh core sample from a well drilled at depth 2700–3500. Basic parameters are shown in Table 1. The samples were dried at 65 °C for 12 h before the experiment until the mass remained unchanged. Sample porosity was measured by helium porosimeter (KXD-III type, Hua’an Co, Ltd., Jiangsu, China). Sample pulse-decay permeability was determined by an ultra-low permeability measurement instrument, confining pressure exerted by water and pore pressure exerted by helium. Test conditions: temperature was 25 °C; confining pressure was 8 MPa; and pore pressure was 5 MPa. These rocks generally have low permeability. The average porosity is 3.28%. The average permeability is 0.0035 mD. The characteristic parameters of the samples are found in Table 1. To be noticed, the focus of this manuscript is to discuss the connection about the imbibition potential and pore structures, thus, the heterogeneous of the rock is ignored. Besides, the direction of cylindrical rocks will not influence the imbibition potential and pore structures of the matrix.

**Figure 3:**
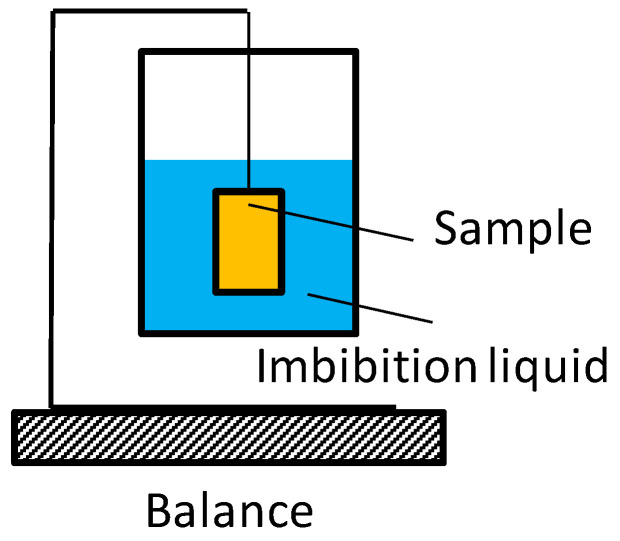
The schematic of the imbibition apparatus.

To uncover the connection between pore size distribution and spontaneous imbibition characteristics, several experimental tests have been conducted. The first one is spontaneous imbibition test. Figure 3 shows the counter-current imbibition equipment. It mainly includes an electronic balance and a data recorder. The electronic balance has an accuracy of 0.0001 g, and it measures the mass of liquid imbibed in cylinder core samples. These samples are submerged in deionized water with all faces open. The computer records the data of mass and time during the imbibition process. The experiments were performed at room pressure (0.1 MPa) and temperature (25 °C). The detailed processes can be found in our pervious works [31].

Tight matrix has abundant nano-micro pores. To get pore size distribution, nitrogen adsorption-desorption experiment is conducted. The endings cut from samples in core preparation process are smashed by jaw crusher first, with debris at diameter 1–3 mm. Then samples are further smashed by a ball grinder and sifted into 40 mesh. The environment temperature 77 K. Detailed analysis is shown in experiment results parts.

## 5. Experiment Results and Data Processing

In this section, spontaneous imbibition results are first shown to observe the diffusion ability for all samples. Second, the pore size distribution of each core is determined by conducting a nitrogen adsorption-desorption experiment. Finally, the fractal imbibition potential model is verified by the test results. The actual pore size distributions are expressed and matched by fractal structure parameters from the numerical model discussed in Section 3. The calculation results of diffusion ability coincide with experimental results.

**Figure 4:**
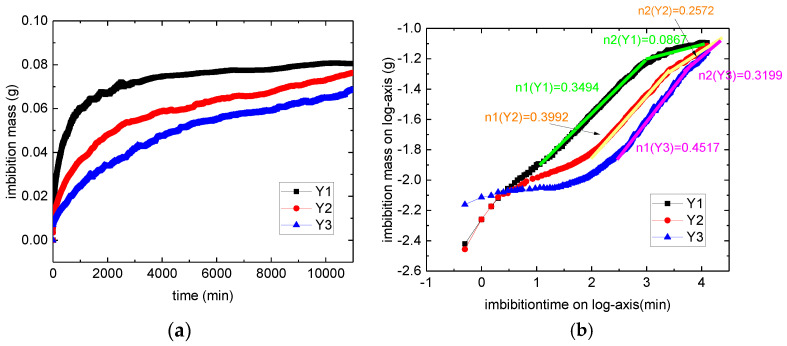
Imbibition curves for samples in regular and log-log coordinates. (**a**) is the regular coordinates, (**b**) is log-log coordinates. The slopes of linear part are marked in this figure.

The spontaneous imbibition is a test using an auto metering system. Imbibition curves of Y1 to Y3 samples are shown in Figure 4. As liquid imbibed in samples, the weight of core increases. In log-log coordinates, there are two liner part on imbibition curves. The slope is called imbibition index. Calculation result is shown in Table 2. The slop of curve can mainly describe as three parts: initial part, capillary part, and diffusion part. Initial part is commonly recognized as influenced by big cracks in core, not every core has this part. Capillary part shows liquid imbibed in cores by capillary force. For tight matrix, the slop is smaller than 0.5, the slop of capillary part varies from 0.35 to 0.45. While in diffusion part, samples with low diffuse ability cannot imbibe more liquids, so for the low-diffuse-ability samples, the slop is smaller. In Figure 4, diffuse ability of Y1 is weak, while Y3 is strong. We also calculated *F_d_* for Y1, Y2, and Y3. The turning point from capillary part to diffusion part is calculated by the crossover point of slop in Figure 4b. The result shows that, the diffuse ability or imbibition potential ranking lists from the highest to lowest is Y3 > Y2 > Y1, which is in accordance with imbibition index result.

**Figure 5:**
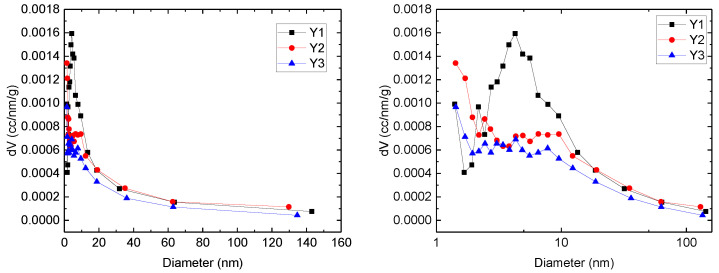
Pore size distribution curves derived by Nitrogen adsorption-desorption tests.

Nitrogen adsorption-desorption experiment is conducted to measure the pore size distribution. Within the environment temperature 77 K, as pressure increases, liquid nitrogen first adhered to the smallest pores then gradually adhered to larger pores. Based on adsorption/desorption curve, pore size distribution is calculated. The explanation result by BJH method is shown in Figure 5. The left figure is on rectangular coordinate system, while right one is on Semi logarithmic coordinate system. Here we can derive that for sample Y1, the main pore size is about 5 nm, much larger than Y2 and Y3. The heterogeneity of Y1 is weaker than that of Y2 and Y3 because the pore scale range of Y2 and Y3 is wider. These characteristics influence the imbibition curve.

## 6. Applications of the Model

In the imbibition network model, fractal parameters of network can be calculated based on the real pore size distribution simulation process, and diffuse ability of model are calculated and compared. The degree of diffuse ability can be verified by imbibition experiment result.

**Figure 6:**
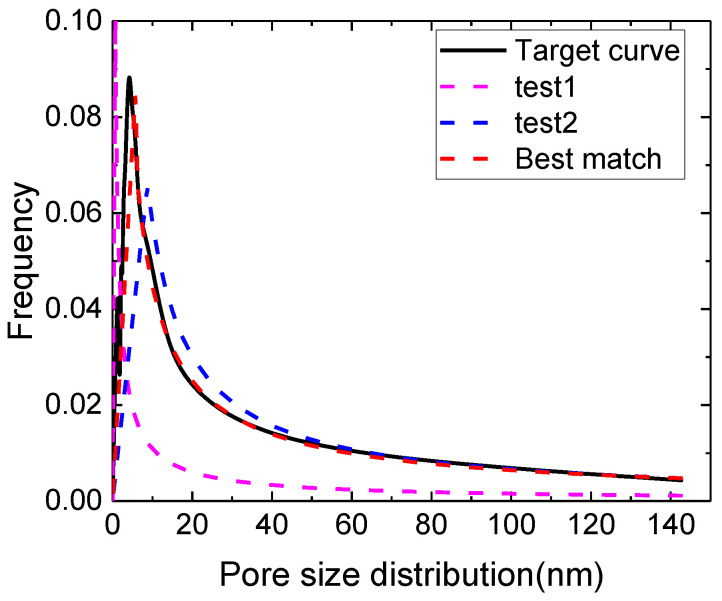
Pore size distribution simulation process.

During the simulation process, we obtained real pore size distribution from nitrogen adsorption curve first. Then, pore size distribution of network model is drawn to compare with the real pore size distribution in the same coordinate. By adjusting fractal parameters of network, the simulated pore size distribution can match the real one. Then the fractal parameters of network can be derived. Figure 6 shows a simple process about how this fitting process works. From the experiment, we gain the pore size distribution (Taking the Y1 sample for an example), which is our target curve. Then we test a parameter combination with $D_{d}$ = 2.6, $D_{l}$ = 1.48, *n* = 2.0 as test 1, and gain the derived pore size distribution, shown as purple curve. Then by changing another parameter combination with test 2 ($D_{d}$ = 2.9, $D_{l}$ = 1.48, *n* = 1.5), we gain the blue curve. After several tests, we gain the best match curve, with parameter combination $D_{d}$ =2.6, $D_{l}$ =1.48, *n* = 1.5. So this is the best match fractal parameters for Y1 sample, listed in Table 3. Generally, in the numerical simulation model, length fractal dimension $D_{l}$, diameter fractal dimension $D_{d}$, and branch number n can be calculated in the matching process. As shown in Figure 6, these three parameters can control the shape of fractal model’s pore size distribution simulation. With the same method, the fractal parameters for Y2 and Y3 can be calculated. The results are listed in Table 3. *k* = 20 in this model based on diameter of biggest pores and smallest pores. From result of dimensionless imbibition potential $Q_{k}{}^{+}$, we can denote that Y2 and Y3 has stronger diffuse ability than Y1, this result matches well with the spontaneous imbibition test’s result in Section 5 and Table 2.

**Figure 7:**
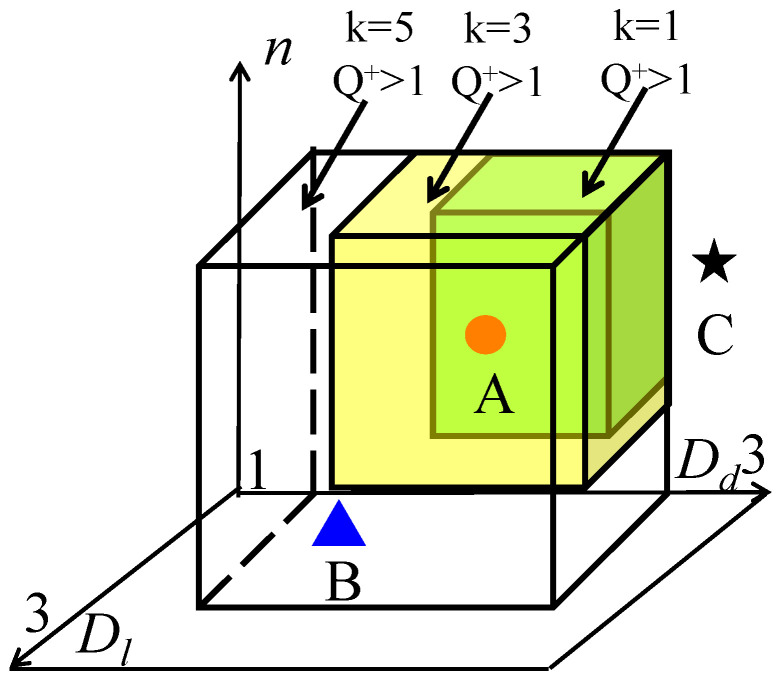
Schematic of imbibition parameter cube.

To clarify the most potential parameter combination for imbibition, we established an imbibition parameter cube based on Equation (8), shown in Figure 7. We mainly focused on the influence of four major factors $D_{l}$, $D_{d}$, n, and k on imbibition potential. So, these four structure parameters have taken into consideration: $D_{l}$, $D_{d}$, n, and k from analytic expression of dimensionless parameter $Q_{k}{}^{+}$ in Equation (8). The X, Y, Z coordinate represents $D_{l}$, $D_{d}$, n. The three parameters can just form a cube model. Any point in this space is presented as the imbibition potential calculated by the combination of the three parameters. Thus, in this cube model, we can easily see which parameter combination will gain the higher imbibition potential $Q_{k}{}^{+}$. k can be regarded as a time-related parameter because as k increases, the imbibition front imbibed in deeper network, and the imbibition time increase. This cube shows the location of the optimum combination of parameters (Figure 7). When k is stable, the parameter combination that leads to the largest $Q_{k}{}^{+}$ in space is defined as the optimum combination of parameters, whereas the parameter combination that leads to ${Q_{k}}^{+}>1$ in space is defined as a good combination of parameters. In Figure 7, zone A denotes that $Q_{k}{}^{+}$ is always bigger than 1 regardless of the changes in k; zone B denotes that $Q_{k}{}^{+}$ may become larger than 1 when k increases; and zone C denotes that $Q_{k}{}^{+}$ will never be larger than 1 regardless of the changes in k. If a parameter combination of a sample falls in zone A, this sample’s pore structure has an excellent imbibition effect. If a parameter combination of one specific sample falls in zone B, this sample’s pore structure may have a potential imbibition effect in the future. If a parameter combination of one specific sample falls in zone C, this sample’s pore structure has a weak imbibition effect. In sum, when we obtain the structure parameter of a sample, we can directly find the imbibition characteristics of this sample from the structure. This technique is convenient. The structure parameter of a sample can be calculated by conducting the following steps. First, we obtain the pore size distribution from the mercury intrusion experiment or the digital core. Then, we can confirm the structure parameters in this model when the pore size distribution in the model matches that in the actual rock. Finally, the imbibition characteristic is shown in this cube.

**Figure 8:**
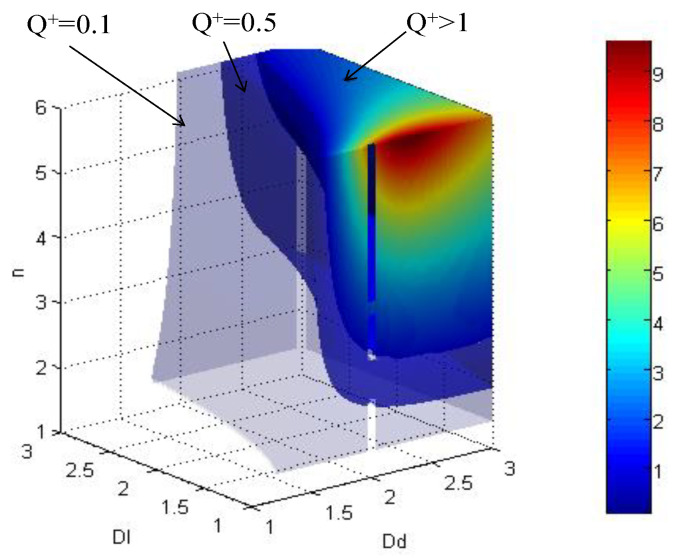
Imbibition parameter cube of fractal network system.

Based on this cube, we calculate the imbibition parameter cube of the model (Figure 8) based on Equation (8). This space contains every $Q_{k}{}^{+}$ under the boundary conditions of $1<D_{l}<3$,$1<D_{d}<3$ and 1<n<6 when k=5. The volume of $Q_{k}{}^{+}>1$ suggests that this model has a good combination of parameters. This volume takes about 1/8–1/6 of the space. In other words, according to the structure parameter, only 20% of parameter combination is good for imbibition. The max $Q_{k}{}^{+}$ is reached under the conditions of a smaller $D_{l}$, a larger n, and an appropriate $D_{d}$.

**Figure 9:**
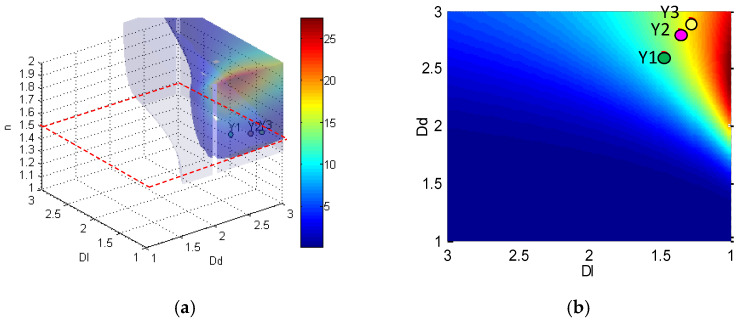
Application of imbibition parameter cube. (**a**) The position of three samples in 3D space. (**b**) The projection of three samples in n = 1.5 plane.

Based on the pore size distribution simulation, we have derived the fractal parameters for the three samples, shown in Table 3. Based on these parameter combination, we can easily draw the imbibition potential points in cube space, shown in Figure 9a,b is the 2D section with *n* = 1.5 for 3D cube, to show the position for Y1, Y2, and Y3 clearly (noticed that for Y3, *n* = 1.51, so the point is a projection on *n* = 1.5 surface). It is clear that all of the sample points fall in the zone A, indicating that these three samples have excellent imbibition potential. Among these three samples, as we calculated in Table 3, the dimensionless imbibition potential $Q_{k}{}^{+}$ list from the strong to weak is Y3 > Y2 > Y1, indicating that Y3 sample has the highest diffusion potential, and Y1 has the lowest diffusion potential. This result matches well with the experiment result in Section 5 and Table 2. But in this model. In this way, we can see the model is a good model to evaluate the diffusion potential.

Generally, in this model, the influences of pore structure parameters, including diameter ratio, length ratio, and branching number, on imbibition rate are examined. The results showed that the imbibition rate decreased as the liquid imbibed into sub-throats. When the pore structure has more branches, larger and shorter sub-throats are likelier to gain a high imbibition rate. The branching level k is critical for the imbibition potential, because a larger branching level extends imbibition time, thus giving more time for the liquid to imbibe into sub-throats and show the imbibition potential of the pore structure.

Pore size distribution greatly influences the imbibition process. Smaller pores and week heterogeneity gain faster imbibition speed, suggesting that these structures have a strong diffuse ability to unlock blocked gas. Thus, imbibition effect should be considered in tight formations. In addition, parameters influenced by pore size distribution, such as imbibition index, imbibition rate, length ratio α, and diameter ratio β, are suitable for representing the imbibition process. Pore structures having a smaller β or a larger α in the fractal model are likelier to gain diffuse ability.

A 3D imbibition parameter cube is constructed to evaluate the percentage of good parameter combinations in all available combinations. Approximately 16% of parameter combinations are good. These good parameter combinations are in the boundary of $D_{d}>1.5$, *n* > 3, and $1<D_{d}<2$. Moreover, the range of good parameter combinations increases as the imbibition front moves forward. This cube is helpful for evaluating the imbibition feature of a pore structure.

This model is a useful tool in evaluating the imbibition potential and understand the connection between pore structure and imbibition potential. Also, it has some promising applications on gas production evaluation for tight reservoir. (1) One is to evaluate the self-unblocking ability for tight matrix. Commonly, during gas production process for a tight matrix, the formation will be fractured by fracturing liquids. After fracturing, some liquids will block the main channels/micro fractures for gas production. By using this model, the imbibition potential of the matrix can be evaluated. For high imbibition potential matrix, the blocked liquid in micro fractures is easier to be imbibed in matrix, thus, this formation is promising. (2) Another application is to evaluate the influence of imbibition on gas production. By using this model, we will understand which kind of pore structure has a higher imbibition potential. Then, during gas production process, the matrix with higher imbibition potential is easier to absorb fracturing liquids, thus, to increase pore pressure and to displace the gas spontaneously by capillary forces. Finally, the spontaneous imbibition may improve the gas production.

## 7. Conclusions

In this work, we generally focus on the influence of pore structure on imbibition potential for a tight matrix. Based on the tree-like pore network model, the dimensionless parameter $Q_{k}{}^{+}$ and its analytical solutions are provided to evaluate the imbibition potential. Additionally, this model is used to evaluate the imbibition potential for three samples. The result matches well with the spontaneous imbibition experiment. Finally, to visualize the influence of parameter combination, we provided the imbibition parameter cube. In this cube, it is easy to evaluate which parameter combination has an excellent imbibition potential. Finally, this cube is used to evaluate the given three tight samples as a simple application. Based on the model, we find that the parameter combination follows the following three characteristics is easier to gain higher imbibition potential: (a) With large number of tiny branches and larger branching levels (higher k); (b) with smaller fractal dimensions on length $D_{l}$ (approaches to 1); (c) with appropriate fractal dimensions on diameter $D_{d}$ (commonly within the range of 2.5–3). Generally, this model is helpful to understand the connection between pore structure and imbibition potential. Also, it provides a promising tool to fast evaluate the imbibition potential of tight matrix.

## Figures and Tables

**Table 1:** Basic property of rocks.

Core Number	Diameter (cm)	Length (cm)	Volume (g/cm^3^)	Porosity (%)	Permeability (mD)
Y1	2.5	0.58	11.45	3.94	0.0039
Y2	2.5	0.57	11.26	2.89	0.0037
Y3	2.5	0.61	11.94	3.01	0.0029

**Table 2:** Slopes and typical points of imbibition curves.

	$n_{1}$	$n_{2}$	Turning Time (min)	$M_{d}(g)$	$M_{max}(g)$	$F_{d}$
Y1	0.3494	0.0867	1110	0.06026	0.08062	0.25
Y2	0.3992	0.2575	2954	0.05248	0.07142	0.27
Y3	0.4517	0.3199	3564	0.04466	0.07672	0.42

**Table 3:** Fractal results based on pore size distribution simulation.

	$D_{d}$	$D_{l}$	*n*	$Q_{k}{}^{+}$
Y1	2.60	1.48	1.50	0.89
Y2	2.80	1.36	1.50	1.15
Y3	2.90	1.28	1.51	1.35
